# 

**Published:** 1891-01

**Authors:** 


VOL. XII.] PUBLISHED MONTHLY AT THREE DOLLARS A YEAR. SINGLE COPIES, THIRTY CENTS. [NO. 1.

(86)

### THE JOURNAL OF COMPARATIVE MEDICINE AND VETERINARY ARCHIVES.

EDITED BY

W. A. CONKLIN, Ph. D., D. V. S.,

*Director of Zoological Gardens, New York.*

RUSH SHIPPEN HUIDEKOPER, M.D., Veterinarian, (*Alfort.*)

*Professor of Sanitary Medicine and Veterinary Jurisprudence, American Veterinary College, New York.*

SURGEON GEN'L'S OFFICE LIBRARY

JANUARY, 1891.

A. L. HUMMEL, M.D., PUBLISHER,

Philadelphia, Pa.

LONDON: BALLIERE, TINDALL & COX.

